# A Great National Duty

**Published:** 1920-02-21

**Authors:** 


### TREATMENT OF INCIPIENT MENTAL DISEASE.

### A Great National Duty.

To urge reform in our methods of dealing with early mental disorder would seem to labour the obvious. The most elementary thinker must long have realised the hopelessness of our system. Unfortunates who fail to conform to the common mental type have hitherto been left to struggle alone at the mercy of chance surroundings. Even though the sufferer desire advice and treatment, he has had nowhere to turn for expert guidance—unless he would risk the stigma of "insanity" or "lunacy." By the time he has qualified for admission to the recognised institutions his case has usually become too desperate for hope of cure. Before the war this policy, representing uneducated public opinion countenanced by governments devoid of scientific outlook, was distinctly cruel and uneconomical: it was also suicidal, and therefore there is now a chance of focussing public opinion upon it.

It is, therefore, with renewed, though guarded, optimism that we read the authoritative communication of mental experts to the *Times*, and the leading article in the same issue (February 6, 1920), and pray that their efforts may meet with success. They emphasise the urgent need of reform. The insane are still dealt with under the provisions of the Lunacy Act, 1890. Although this Act fulfils the important functions of protecting society and safeguarding the liberty of the subject, it affords lamentably insufficient scope for the treatment of the patient.

### The Revision Recommended.

The Medico-Psychological Association of Great Britain and Ireland has recommended revision of our methods of treating incipient mental diseases and the provision of opportunity for study and research. The early symptoms of disorder occur long before certification is possible, at a time when the need for well-considered treatment is obviously urgent, as it is only then likely to be successful. On the other hand, facilities for skilled treatment at this early stage have been deplorably few.

For this state of affairs an ignorant public is to blame for stigmatising the mentally afflicted, in whatsoever category or degree, as almost culpably beyond the pale—an opinion which has been regrettably supported by the official attitude.

A short amending Bill to the Lunacy Act would provide for treatment in the early and curable stages of mental disorder without certification, by embodying the reforms most urgently needed. The proposals outlined by the sub-committee of the Medico-Psychological Association are, in brief, as follows: "The provision of clinics—the so-called psychiatric clinics—in large centres of population, and especially in connection with the general hospitals, and where schools of medicine exist; the extension of the system of voluntary admission (which now obtains in respect of licensed houses and registered hospitals for the insane), so that patients, whether of the private or rate-aided class, may place themselves for treatment in county borough mental hospitals; or further provision for the private patient class, so that, with the approval of the Board of Control, such may be received without certification (but with the cognizance of the central authority) into homes, privately-owned or supported wholly or partly by voluntary contributions, and also into existing public and private mental hospitals ('licensed houses'); also received, with the sanction of the board, as single patients, without certification, provided that a medical practitioner gives a written recommendation, stating that suitable treatment can be obtained in the proposed house.

"Of the above proposals, that concerned with the establishment of clinics in psychiatry—with in-patient and out-patient departments—as an integral part of the general hospital system is the most important; and in the operation of these clinics lies our main hope of avoiding the never-ending extensions to existing asylums."

### Means Already at Hand.

Several clinics of this nature were already in existence before the war and have done excellent work in the direction of treatment, teaching, and research. Indirectly, the war has been instrumental in establishing others: but they have been insufficient to cope with its added burden. Incidentally, the war has served to make it clear, even to the most unscientific observer, that mental disorder is not entirely due to inherent defect or to "weakness of will"—and one cannot over emphasise the importance of the public attitude in this matter. During the war, moreover, soldiers were received and treated in mental hospitals without orders or certificates for at least nine months before being sent to asylums. This procedure had such tremendous advantages as to demonstrate incontrovertibly the desirability of applying a similar system to civil life.

Though the reforms outlined have been repeatedly urged and presumably recognised by the Government, there is no tangible evidence that any step has been taken to put them into practice. Truly, as the *Times* leader states, "a point has been reached where legislation falls behind scientific understanding." Legislation always will fall behind: but we may at least expect of our legislators that they should keep in sight.

We would add one note of warning: "Scientific understanding" should be represented by those alone who are qualified to express its judgments. Nowadays the number of psychological amateurs, within and without the medical profession, is legion. At a time when a united front is essential their fruitless controversies ever and again confuse the issue.

